# Double Trouble: A Rare Case of Synchronous Breast and Thyroid Carcinomas

**DOI:** 10.7759/cureus.65256

**Published:** 2024-07-24

**Authors:** Anuradha S Dnyanmote, Himashree M P, Sandeep Kumar, Kinjal Vasava

**Affiliations:** 1 Department of General Surgery, Dr. D.Y. Patil Medical College, Hospital and Research Centre, Pune, IND

**Keywords:** papillary carcinoma of thyroid, mucinous carcinoma of breast, breast and thyroid malignancy, double primary tumors, synchronous carcinoma

## Abstract

Breast carcinoma and thyroid carcinoma are among the most common cancers affecting women. Although it is rare to encounter synchronous primary tumors of the thyroid and breast in clinical practice, the incidence of both differentiated thyroid and breast cancers has significantly risen over the last 20 years. Despite having a lower mortality risk compared to other types of cancer, managing a dual diagnosis of these malignancies poses unique challenges and requires a thorough evaluation and strategic treatment plan.

Here, we report a rare case of double primary malignancy of the breast and thyroid in a 59-year-old female who presented with complaints of a lump in the left breast, along with an incidental finding of thyroid swelling, which had conflicting findings in various preliminary evaluations. In this reported case, the patient underwent a total thyroidectomy based on a frozen section report suggestive of papillary carcinoma along with a modified radical mastectomy because of mucinous carcinoma of the left breast, which by itself is a rarity. This constituted a great challenge in managing both malignancies simultaneously.

In conclusion, synchronous breast and thyroid carcinomas constitute an atypical clinical scenario that requires detailed evaluation and a multidisciplinary management approach. Further research is needed to understand this condition's underlying pathophysiology and genetic background to improve therapeutic outcomes for affected individuals.

## Introduction

"Synchronous" tumors refer to cases where a second primary malignancy is identified within six months of the initial cancer diagnosis, while "metachronous" tumors are diagnosed more than six months after the first primary malignancy. According to established guidelines, the incidence of a second primary malignancy in general in cancer patients is approximately 10% [1]; however, the occurrence of synchronous neoplasms of the thyroid and breast is notably rare. Each tumor must be distinct in its microscopic and morphological features, ensuring that neither arises as a metastasis from the other. The first known case of this dual malignancy was reported by Billroth in 1889 [2]. In Western India, the incidence of concurrent primary tumors of the breast and thyroid is 1.51%, and an increasing trend in thyroid cancer cases associated with breast malignancies has been noted [3,4].

In this report, we discuss a case where both breast carcinoma and thyroid carcinoma were diagnosed concurrently. The breast carcinoma observed was of the mucinous type [5], which is uncommon and generally has a more favorable prognosis if diagnosed early compared to breast carcinoma of the not otherwise specified type (No. type), and the thyroid carcinoma was of the papillary type [5].

Clinically, patients might present with a variety of symptoms. For instance, an asymptomatic thyroid nodule may be incidentally discovered during the evaluation for a breast lump, as observed in our case, or patients may exhibit symptomatic thyroid nodules alongside a breast lump. Diagnostic evaluation generally involves a multidisciplinary approach, including ultrasound, mammography, fine needle aspiration cytology (FNAC), and core needle biopsy to characterize both tumors [6-10] accurately. This comprehensive diagnostic process is essential for developing an optimal plan to manage both malignancies simultaneously. The prognosis depends on multiple factors, such as the stage and type of both breast and thyroid malignancies. Long-term surveillance is vital to detecting recurrences or metastases in both cancers.

## Case presentation

A 59-year-old female presented with a complaint of a lump in the left breast persisting for three months. Initially small, the lump increased to a size of 4 cm by 3 cm and was not associated with any history of trauma or pain. There was no history of purulent or bloody nipple discharge, nor were there any similar lumps in the right breast. Additionally, there was no history of fever, no other lumps elsewhere in the body, or no abdominal distension. The patient was nulliparous and had a history of dysfunctional uterine bleeding 20 years ago, for which she was initially managed medically and eventually underwent a hysterectomy.

On examination of the bilateral breasts and axillae, a single hard lump measuring 4 by 3 cm was palpable in the retro-areolar region at the 4 o'clock position in the central quadrant. There were no signs of dimpling, nipple retraction, or skin changes. No lump was palpable in the opposite breast, and no lymphadenopathy was noted in the bilateral axillae. Incidentally, some fullness was observed in the anterior aspect of the neck, despite the patient having no complaints or symptoms related to that. On palpation of the neck, minimal contour irregularity was noted on the left and right sides of the midline in the neck; 4 by 3 cm of swelling was palpated on the anterolateral aspect of the neck bilaterally, firm in consistency, and moving with deglutition.

**Figure 1:**
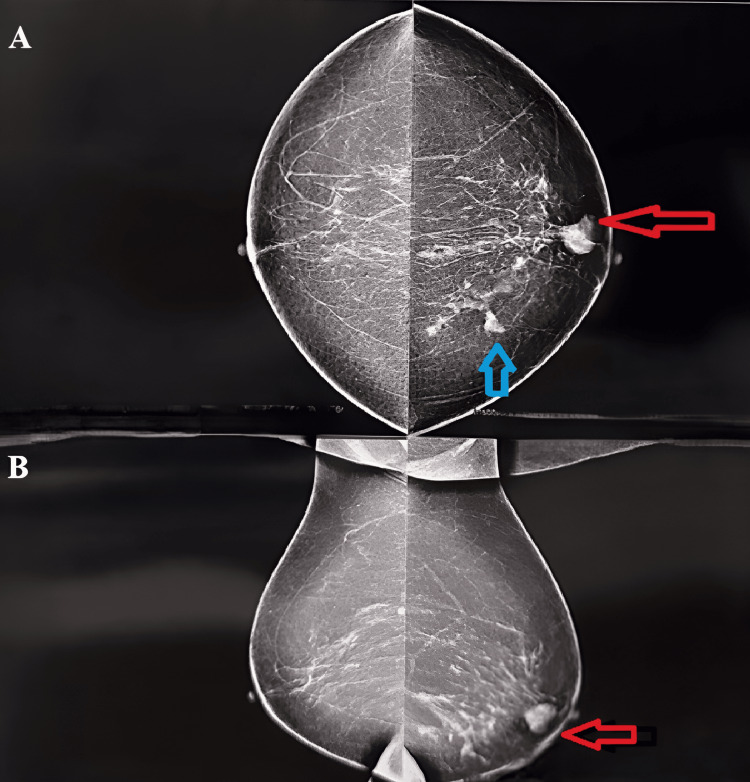
Mammography of bilateral breasts with axillae. Figure 1A is a craniocaudal view, and Figure 1B is a mediolateral oblique view. The red arrow depicts the lesion in the left breast. The blue arrow shows microcalcification around the tumor

Bilateral mammography of the breasts and axilla revealed a lobulated isoechoic to a hyperechoic lesion in the retro-areolar region of the left breast, with a prominent duct and multiple intralesional and intraductal microcalcifications. As per the Breast Imaging Reporting and Data System (BIRADS), a score of 4A was assigned [6] (Figure 1).

An ultrasound of the neck revealed that the thyroid gland was mildly enlarged with heterogeneous echotexture, indicative of thyroiditis. In the left lobe of the thyroid, a well-defined, lobulated, smoothly marginated hyperechoic solid lesion measuring 9 x 21 x 14 mm was observed, and according to the Thyroid Imaging Reporting and Data System (TIRADS), it was classified as TIRADS 3 [7]. Another well-defined, smoothly marginated, hyperechoic solid lesion measuring 20 x 30 x 32 mm was also noted in the left lobe, classified as TIRADS 3. A well-defined, smoothly marginated, mixed hyperechoic solid-cystic lesion measuring 20 x 17 x 17 mm was detected and classified as TI-RADS 4 in the right lobe. A thyroid function test was done, which was within the normal limits, and an anti-thyroid peroxidase antibody (anti-TPO) showed elevated levels [8] (Table 1). Fine needle aspiration cytology (FNAC) from the thyroid suggested colloid goiter. 

**Table 1:** Thyroid function test and anti-TPO antibody observed in patient with reference interval as per ATA guidelines 2017 (American Thyroid Association)

Test description	Observed value	Biological Reference interval
T3 (TOTAL)	0.85	0.64 to 1.52 ng/ml
T4 (TOTAL)	8.49	4.87 to 11.72 µg/dL
TSH	0.71	0.35 to 4.94 µIU/mL
Anti TPO antibody	>600	<5.61 IU/mL

A core needle biopsy of the breast lump indicated mucinous carcinoma of the breast, with immunohistochemistry (IHC) revealing estrogen receptor (ER) positivity, p63 negativity, progesterone receptor (PR) negativity, and human epidermal growth factor 2 (HER 2 NEU) negative status. The Ki-67 proliferative index was 10-15% suggestive of a low index of proliferation [9] (Table 2). The metastatic workup showed no evidence of metastasis. A clinical diagnosis of carcinoma of the breast, staged as T2N0M0, with an undiagnosed benign thyroid condition was considered. 

**Table 2:** Immunohistochemistry panel for breast carcinoma in our patient

IHC	Observed value	Interpretation
ER	3	0: no receptors positive
		1: <25% positive
		2: 25 to 50% positive
		3: >50% positive
PR	0	0: no receptors positive
		1: <25% positive
		2: 25 to 50% positive
		3: >50% positive
HER 2 Neu	0	0 or 1+: negative
		2+: equivocal
		3+: positive
Ki 67 index	10 to15%	<15 %: low expression
		15 to 30%: intermediate expression
		>30 %: high expression

**Figure 2:**
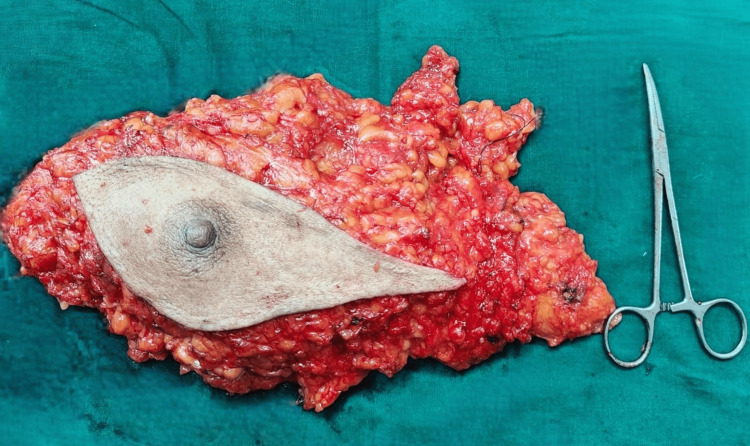
Excised specimen of the left breast

**Figure 3:**
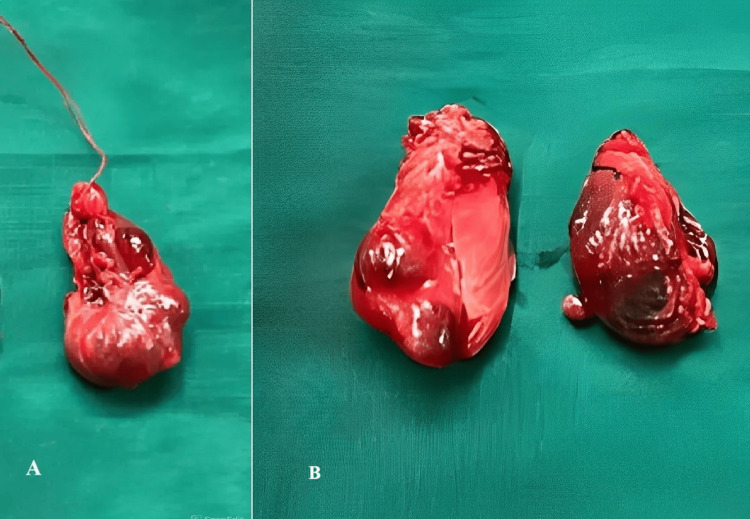
An excised specimen of total thyroidectomy. Figure 3A shows the right enlarged lobe sent for the frozen section. Figure 3B shows the left lobe of the thyroid and the isthmus

Routine blood investigations like a complete blood count, liver function test, renal function test, serum electrolytes, and coagulation profile for presurgical anesthetic fitness were normal. The patient was advised to undergo breast-conserving surgery followed by radiotherapy, along with a frozen biopsy of the right thyroid lobe, with further management to be determined based on the biopsy results for the thyroid. However, the patient declined postoperative radiotherapy, leading to the decision to perform a modified radical mastectomy (MRM). Consequently, a modified radical mastectomy (Figure 2) with a right lobectomy and frozen section analysis was performed. The frozen section was positive for papillary carcinoma of the thyroid (PTC), and hence a total thyroidectomy was performed (Figure 3).

**Figure 4:**
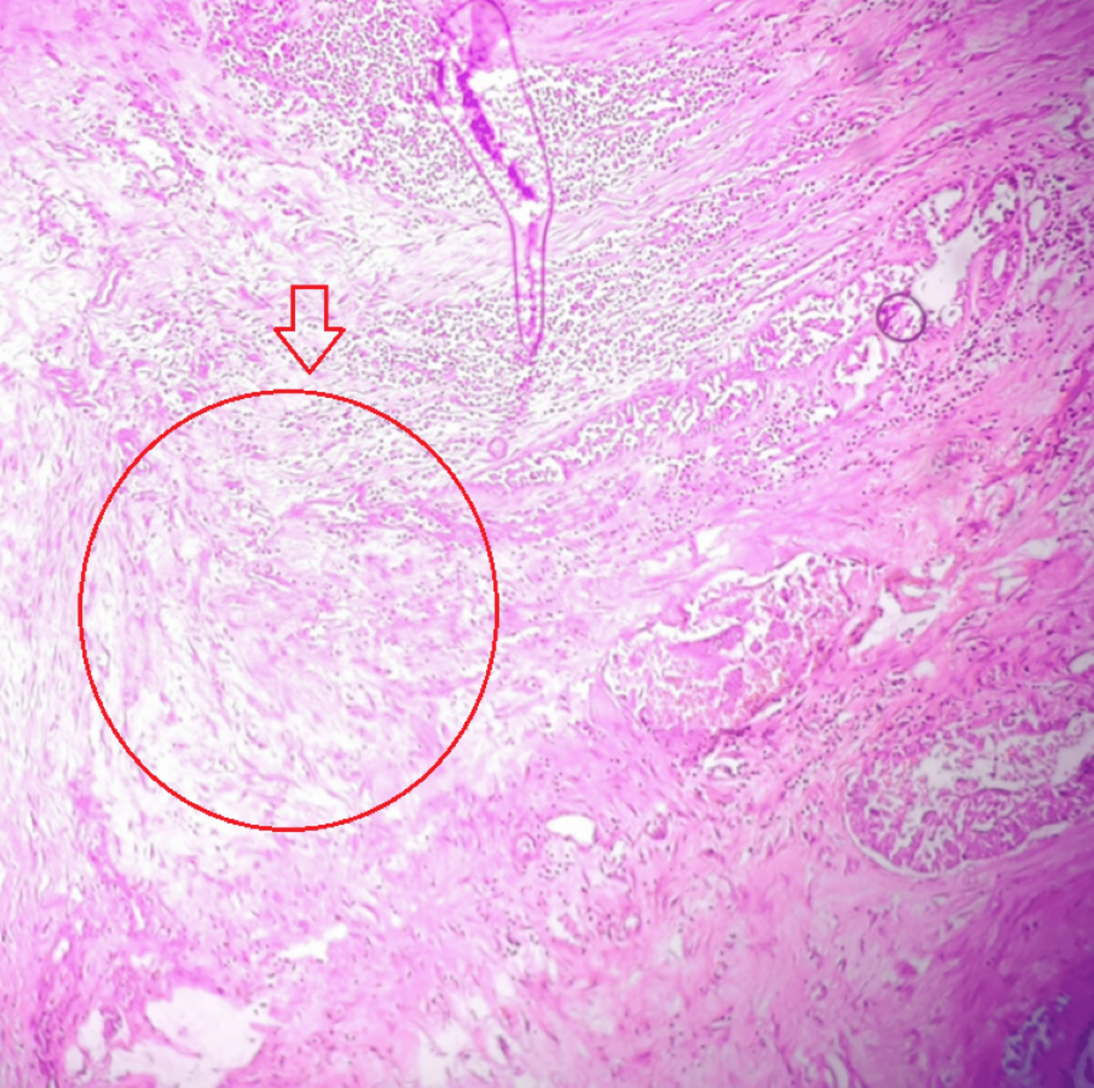
Shows features of the presence of abundant extracellular mucin, which gives it a gelatinous or colloid-like appearance, as shown with a red arrow. Cells are arranged in clusters, creating a pool of mucin, and appear separate from the rest of the breast tissue, as shown by a red circle

**Figure 5:**
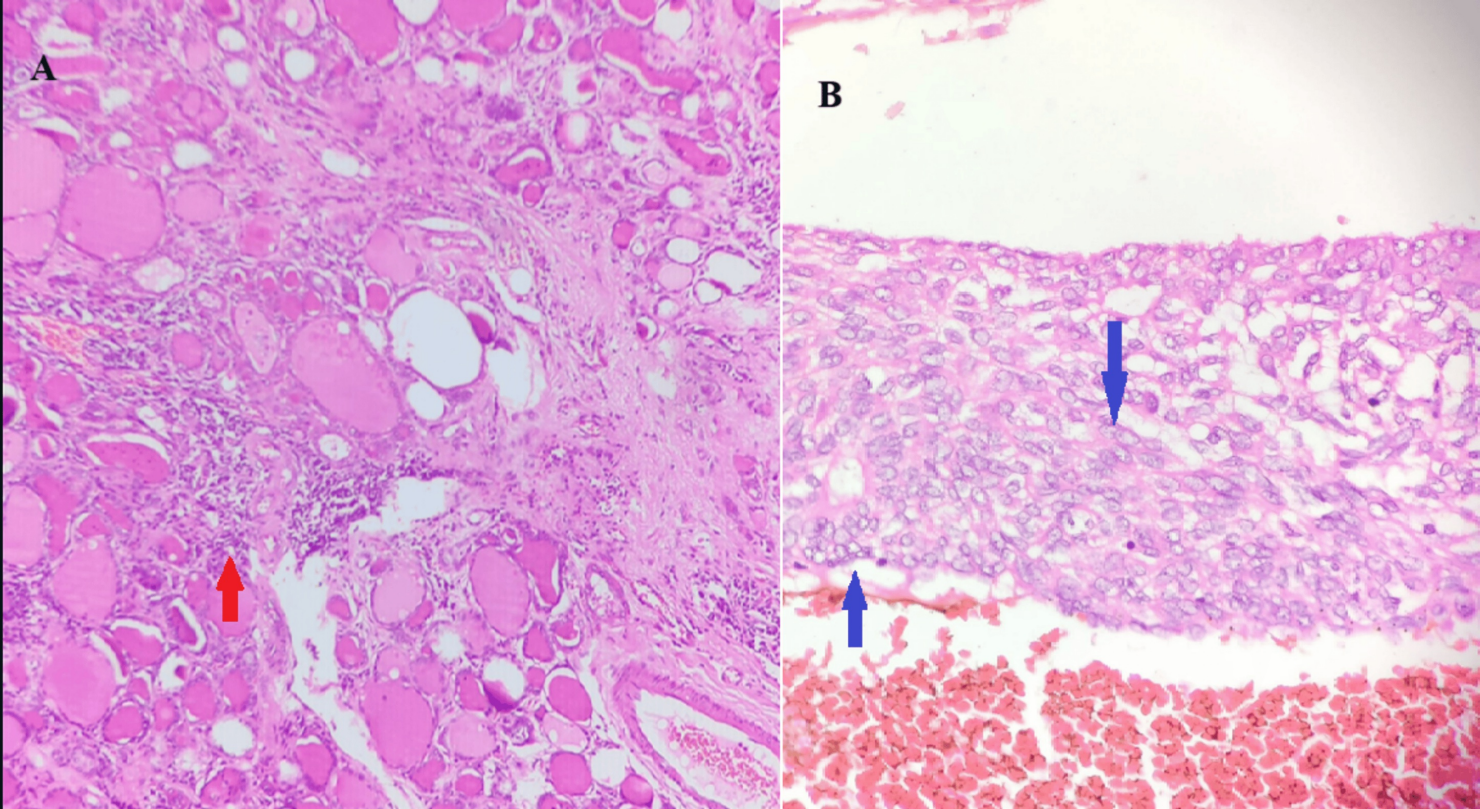
5A: Depicts the vesicular nuclei of thyroid follicles in Hashimoto’s thyroiditis, as shown with red arrows. 5B: Depicts Orphan Annie’s eye nuclei, a classical feature of papillary carcinoma of the thyroid

Post-surgery, the histopathology reports were suggestive of mucinous carcinoma of the left breast and papillary carcinoma of the right lobe of the thyroid, with Hashimoto's thyroiditis predominantly on the left lobe of the thyroid, with a resection margin free of tumors and no lymphovascular invasion in both the breast and thyroid (Figures 4, 5A, 5B).

Following surgery, a medical oncology opinion was sought, and the patient was started on hormonal therapy with letrozole 2.5 mg (an aromatase inhibitor) once a day and TSH suppression therapy with levothyroxine 150 mg once a day [10].

## Discussion

Breast and thyroid carcinomas are among the most prevalent cancers in India; both the breast and thyroid glands are endocrine organs, and current research into synchronous primary malignancies of these sites emphasizes the role of thyroid hormones in activating oncogenic pathways. One such pathway involves the mutation of thyroid hormone receptor-β, which also promotes mammary hyperplasia by activating the transcription factor 'signal transducer and activator of transcription 5’ (STAT5) [4]. Some researchers have explored the involvement of thyroid hormones, thyroid peroxidase antibodies, and thyroid Ras-regulating enzymes in the mechanisms underlying synchronous primary tumors [11]. Additionally, some studies have suggested that increased levels of nuclear protein 1, retinoid-inducible nuclear factor, and nuclear receptor coactivator act as potential co-activators of both breast and thyroid malignancies [12,13].

In our case, a diagnosis of mucinous carcinoma of the breast was made. This rare malignancy constitutes 2% of breast cancers and is characterized by extracellular epithelial mucin surrounding neoplastic cells [14]. In comparison with non-specific breast carcinoma, mucinous breast carcinoma has a favorable prognosis and excellent long-term survival rates, attributed to a lower incidence of nodal involvement, favorable histological grading, and high expression levels of ER and PR, hence paving the way for hormonal therapy as adjuvant treatment [15]. In our case, the patient has been started on hormonal therapy post-surgery. Considering the age of 59 years and postmenopausal status, our patient is starting on Letrozole. Some articles have mentioned that mucinous carcinomas are mostly HER-2 negative; in our case, also HER-2 receptors are negative [16]. One study has also reported that pure mucinous carcinoma and mixed variant mucinous carcinoma both mostly fall under the luminal A type and luminal B type, respectively, and this difference is due to more genetic instability in the mixed variant than the pure variant [17]. In our case, considering the estrogen receptor-positive status and human epidermal growth factor receptor-2 negative status, this mucinous variety can be attributed to the luminal A type.

Mucinous carcinoma shows very low rates of lymphatic spread, ranging from 12-14%, with a negligible spread in the pure mucinous variety compared to mixed mucinous carcinoma [18]. Five-year disease-free survival rates range from 81% to 94%, reaching the higher end when lymph nodes are negative [14]. In invasive ductal carcinoma, particularly with multiple duct involvement, nodal status has been identified as a significant predictor of distant disease-free survival [19]. Some articles have also published that pure mucinous breast carcinoma can be omitted from adjuvant chemotherapy since it has a better prognosis than invasive ductal carcinoma not otherwise specified type (NOS). Similarly, in our case, we are planning not to give chemotherapy considering the early tumor (T) stage and the mucinous variety [20]. Some articles have also published that pure mucinous and tumor size less than 5 cm are independent favorable risk factors; in our case, both of these favorable factors are present [21].

Although late distant metastases can occur in pure mucinous carcinoma as per previous literature, tumor size is an independent prognostic factor, though less significant than nodal status [22]. Other studies suggest that tumor size does not significantly impact prognosis or survival, as the majority of the tumor volume is mucin [23]. The overall size of the breast lesion in our case falls under T2, with lots of mucin pool, as shown in the histopathology imaging above.

Elevated anti-TPO antibodies have been noted in some of these synchronous malignancy patients. In many published articles, it is proven that there is a protective role for mammary carcinoma by preventing early metastasis and recurrence, and a more indolent course for mammary carcinoma is noted among the anti-TPO positive cases [24]. In our case, the patient, who initially presented to the hospital with a primary complaint of mammary carcinoma, tested positive for anti-TPO antibody, with the breast lesion measuring less than 5 cm (T2) and no metastasis, as mentioned in previously published articles.

The sodium iodide symporter (NIS), essential for iodide transport in thyrocytes, is also found in lactating breast and breast cancer cells. NIS expression is predominantly seen in ER-positive and also in some triple-negative breast cancer subtypes, and the molecular mechanisms affecting NIS have relocated it from the cytoplasmic location to the plasma membrane, unlike in the lactating breast [25]. In our case, we have done a routine immunohistochemistry panel without NIS, but further studies or inclusion of NIS in the immunohistochemistry panel for breast cancer must be decided upon. In our case, as discussed earlier, the histopathology report was suggestive of mucinous carcinoma, with a positive IHC for the estrogen receptor and a negative for the rest.

Studies have suggested that breast tissue contains receptors for thyroid-stimulating hormone (TSH). Along with estrogen and progesterone receptors in the mammary gland, this may have a role in breast cancer initiation and progression [26]. Studies have also suggested that elevated TSH levels have been associated with both primary thyroid and breast malignancies, contributing to our understanding of the occurrence of synchronous thyroid and breast malignancies [27]. In our case, the estrogen receptor is positive, but the TSH levels were within the normal limits, despite being a synchronous malignancy.

Papillary thyroid carcinoma is the most common thyroid neoplasm, characterized by follicular cell differentiation and distinct nuclear features like the orphan Annie eye nucleus with nucleolar grooving, and generally carries a favorable prognosis [28]. Active surveillance is appropriate for managing low-risk PTC with a size less than 1 cm, involving biannual high-quality ultrasound examinations of the neck. Surgery is recommended if there is a 3 mm increase in axial dimension or a greater than 50% increase in tumor volume with a short doubling time [29]. Following thyroidectomy, patients require lifelong thyroid hormone replacement therapy, typically with levothyroxine (LT4) monotherapy. Since TSH can stimulate residual PTC cells if it is present in the tumor bed, the initial LT4 dosage should be sufficient to suppress TSH levels. A thyroid function test along with thyroglobulin (TG) should be reassessed 6 to 8 weeks post-initiation of LT4 therapy, with dosing adjustments based on the findings. The use of TSH suppression therapy should be carefully evaluated due to the potential risk of complications associated with the high dose of thyroxine. In our case, we have started the patient on a suppressive dose of thyroxine post-surgery.

Some articles say that in cases of synchronous detection of PTC and breast ductal carcinoma, it is advised to prioritize the resection of breast ductal carcinoma first, since it has a higher propensity to metastasis and a poorer prognosis than thyroid carcinoma, if at all thyroid carcinoma is not an anaplastic or medullary variant [30,31]. In our case, we have proceeded with surgery at both sites in the same sitting, considering the general well-being and anesthetic fitness of the patient.

## Conclusions

In conclusion, synchronous primary tumors of the breast and thyroid are extremely rare. It can be sporadic or syndromic, like in Cowden syndrome, where genetics play a role. This case report and review highlight the possibility of concurrent breast and thyroid cancers, particularly the possibility of a histopathological type of breast carcinoma other than the invasive ductal carcinoma NOS type. This report also briefs on the treatment challenges and the need for a multidisciplinary approach to managing such rare synchronous presentations of a specific histological variety.
